# Genetic variants and risk of gastric cancer: a pathway analysis of a genome-wide association study

**DOI:** 10.1186/s40064-015-1005-8

**Published:** 2015-05-06

**Authors:** Ju-Han Lee, Younghye Kim, Jung-Woo Choi, Young-Sik Kim

**Affiliations:** Department of Pathology, Korea University Ansan Hospital, 123, Jeokgeum-Ro, Danwon-Gu, Ansan-Si, Gyeonggi-Do 425-707 Republic of Korea

**Keywords:** Genome-wide association study, Pathway-based analysis, Gastric cancer

## Abstract

This study aimed to discover candidate single nucleotide polymorphisms (SNPs) for hypothesizing significant biological pathways of gastric cancer (GC). We performed an Identify Candidate Causal SNPs and Pathways (ICSNPathway) analysis using a GC genome-wide association study (GWAS) dataset, including 472,342 SNPs in 2,240 GC cases and 3,302 controls of Asian ethnicity. By integrating linkage disequilibrium analysis, functional SNP annotation, and pathway-based analysis, seven candidate SNPs, four genes and 12 pathways were selected. The ICSNPathway analysis produced 4 hypothetical mechanisms of GC: (1) rs4745 and rs12904 → *EFNA1* → ephrin receptor binding; (2) rs1801019 → *UMPS* → drug and pyrimidine metabolism; (3) rs364897 → *GBA* → cyanoamino acid metabolism; and (4) rs11187870, rs2274223, and rs3765524 → *PLCE1* → lipid biosynthetic process, regulation of cell growth, and cation homeostasis. This pathway analysis using GWAS dataset suggests that the 4 hypothetical biological mechanisms might contribute to GC susceptibility.

## Introduction

Despite a decline in its incidence, gastric cancer (GC) is still the second most common cause of cancer-related death worldwide (Hohenberger and Gretschel 2003). Furthermore, GC remains one of the most prevalent high-mortality cancers in Northeast Asia (Hohenberger and Gretschel 2003). *Helicobacter pylori* infection is the strongest risk factor for GC (Polk and Peek 2010), but only a small proportion of infected individuals develop malignancy. Thus, genetic factors such as polymorphisms in GC-related genes, in addition to dietary factors and environmental factors, substantially contribute to GC susceptibility (Milne et al. 2009).

Genome-wide association studies (GWAS) have proved successful in identifying associations between specific genes and complex diseases (Manolio 2010), and opened a new phase in researching the genetic causes of disease. Furthermore, GWAS datasets are increasingly being used to recognize the biological pathways underlying complex diseases (Ramanan et al. 2012), because the functional pathway analysis using genomic datasets has high statistical power to detect the biological mechanisms of disease causation (Ramanan et al. 2012).

Recently, (Zhang et al. 2011a) developed the pathway analysis tool called Identify Candidate Causal SNPs and Pathways (ICSNPathway) analysis. This method highlights the candidate SNPs and their corresponding candidate pathways from GWAS data by integrating linkage disequilibrium (LD) analysis, functional SNP annotation, and pathway-based analysis (PBA) (Zhang et al. 2011a). The ICSNPathway analysis provides candidate SNPs and their corresponding candidate pathways using GWAS data, thereby making it easier to link variants to biological mechanisms.

We conducted ICSNPathway analysis using a GC GWAS dataset available online to identify candidate SNPs and promising biological mechanisms that contribute to GC susceptibility.

## Methods

### GWAS dataset

The GC GWAS dataset is publicly available from the NCBI dbGap (http://www.ncbi.nlm.nih.gov/gap). The dataset includes genotypes of 472,342 SNPs on Illumina 660 W Quad chip from 2,240 GC cases and 3,302 controls of Chinese ethnicity (Abnet et al. 2010; Li et al. 2013). Study participants were drawn from the Shanxi Upper Gastrointestinal Cancer Genetics Project and the Linxian Nutrition Intervention Trial, which included a total of 1,625 GC cases and 2,100 controls. Six hundred and fifteen GC cases and 1,202 controls from the Shanghai Men’s Health Study, the Shanghai Women’s Health Study, and the Singapore Chinese Health Study were also included in the database. Controls were matched for age (±5 years), sex, and geographical location and they were all cancer-free at the time of enrollment (Abnet et al. 2010; Li et al. 2013). The dataset was filtered to prevent genotyping errors. The SNPs were excluded if they showed a call rate lower than 90% in cases or controls or significant deviation from Hardy-Weinberg equilibrium in the controls (*P* < 10^–4^). Finally, 470,698 SNPs were left for downstream pathway analysis.

### ICSNPathway analysis

We conducted ICSNPathway analysis using the GC GWAS dataset in two-stages (Zhang et al. 2011a). First, candidate causal SNPs were pre-selected by LD analysis and the most significant functional SNPs were annotated. Next, biological mechanisms for the pre-selected candidate causal SNPs were found using PBA. A full list of GWAS SNP *P-*values was used for the ICSNPathway analysis. The ICSNPathway analysis is based on LD analysis and the discovery of functional SNPs using improved-gene set enrichment analysis (*i*-GSEA). The ICSNPathway searches for SNPs in LD with the most significant SNPs in a GWAS dataset to identify more possible candidate causal SNPs based on the extended dataset, such as HapMap data. We selected the optional parameters for LD: Chinese Han in Beijing as the HapMap population; a cut-off for LD measurement of *r*^2^ = 0.8; and a maximum distance to search LD neighborhoods of 200 kb. The ICSNPathway pre-selects candidate causal SNPs based on functional SNPs, which are defined as SNPs that may alter protein, gene expression or the role of protein in the context of the pathway. Functional SNPs include deleterious and non-deleterious, non-synonymous SNPs; SNPs leading to the loss or gain of a stop codon; SNPs resulting in a frame shift; SNPs located at essential splice sites; and SNPs in regulatory regions. The ICSNPathway server detects pathway-associated traits in the full list of GWAS SNP *P-*values using *i*-GSEA.

The term “most significant SNPs” denotes SNPs with a *P-*value below a certain threshold. The *P-*value threshold to extract the most significant SNPs can be specified using the GWAS SNP *P-*values. The ICSNPathway analysis presents the most significant pathways from the original GWAS when a *P-*value threshold less than 1 × 10^−4^ is chosen. Two parameters were set for the analysis. The first parameter was ‘within gene only’ meaning that only the *P-*values of SNPs located within genes were utilized in the PBA algorithm. The second was a false discovery rate (FDR) cut-off of 0.05 for multiple testing corrections. The FDR, which is defined as the expected proportion of false positives among all significant tests, allows researchers to identify a set of positive candidates. We used cut-offs of 5 minimum and 100 maximum in order to avoid overly narrow or overly broad functional categories.

The ICSNPathway analysis used four pathway databases including the Kyoto Encyclopedia of Genes and Genomes (http://www.genome.jp/kegg/pathway.html) (Kanehisa et al. 2010), BioCarta (http://www.biocarta.com/genes/index.asp), Gene Ontology biological process (http://www.geneontology.org) (Ashburner et al. 2000), and Gene Ontology molecular function (http://www.broadinstitute.org/gsea/msigdb/index.jsp). When a candidate SNP was not present on a particular genotyping array, proxy SNPs in LD for that candidate SNP were identified, based on observed LD patterns in HapMap. Therefore, SNP annotation and proxy search (SNAP) was performed, which is a tool for the identification and annotation of proxy SNPs using HapMap.

## Results

**Figure 1:**
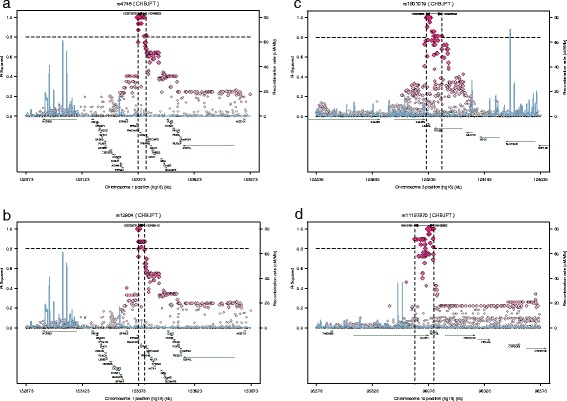
Regional LD plots of rs4745 (*EFNA1*) **(a)**, rs12904 (*EFNA1*) **(b)**, rs1801019 (*UMPS*) **(c)**, and rs11187870 (*PLCE1*) **(d)** SNPs. SNPs are plotted along with proxies (based on HapMap data on Chinese Han in Beijing) as a function of genomic location and are annotated by recombination rate across the locus (*light-blue line*). On the y-axis, pairwise *r* ^2^ values are provided for each proxy SNP using color codes.

Using GWAS SNP *P-*values as inputs, the ICSNPathway analysis identified seven candidate causal SNPs, four genes, and 12 candidate causal pathways (http://ICSNPathway.psych.ac.cn/getResult.do?tag=4904B29B74A51307E8CFD85B4466A802_1374721534621) (Tables 1 and 2) (Figure 1). SNPs rs4745 and rs12904, which were not represented in the original GWAS, are in LD with rs4460629 (r^2^ = 1.0) (-log_10_ (*P*) = 6.472). SNPs rs1801019, rs364897 and rs11187870, which were not represented in the original GWAS, were in LD with rs4234221, rs4460629 and rs3781264, respectively (r^2^ = 0.824, 0.924, 0.857) (-log_10_ (*P*) = 4.087, 6.472, 10.405, respectively). SNPs rs2274223 and rs3765524, which were represented in the original GWAS (-log_10_ (*P*) = 8.633 and 8.556, respectively), were not in LD with any SNP.

**Table 1:** **Candidate causal SNPs and pathways**

**Candidate SNP**	**Functional class**	**Gene**	Candidate causal pathway*	**-log**_**10**_ **(** ***P*** **)†**	**In LD with**	**r** ^**2**^	**D’**	**-log**_**10**_ **(** ***P*** **)‡**
rs4745	Nonsynonymous coding	*EFNA1*	1	-	rs4460629	1.0	1.0	6.472
rs12904	Regulatory region	*EFNA1*	1	-	rs4460629	1.0	1.0	6.472
rs1801019	Nonsynonymous coding	*UMPS*	2,3	-	rs4234221	0.824	1.0	4.087
rs364897	Nonsynonymous coding	*GBA*	4,11	-	rs4460629	0.924	1.0	6.472
rs11187870	Regulatory region	*PLCE1*	5,6,7,8,9,10,12	-	rs3781264	0.857	1.0	10.405
rs2274223	Nonsynonymous coding	*PLCE1*	5,6,7,8,9,10,12	8.633	rs2274223	-	-	8.633
rs3765524	Nonsynonymous coding	*PLCE1*	5,6,7,8,9,10,12	8.556	rs3765524	-	-	8.556

SNP; single nucleotide polymorphism, LD; linkage disequilibrium.

*numbers indicate the indexes of pathway ranked significance (false discovery rate).

†-log_10_ (P) values of candidate causal SNPs in the original genome wide association studies (GWAS), - denotes that this SNP is not represented in the original GWAS.

‡-log_10_ (P) values of SNPs in LD with candidate causal SNPs in the original GWAS.

**Table 2:** **Candidate causal pathways**

**Index**	**Candidate causal pathway**	**Description**	**Nominal** ***P***	**FDR**
1	Ephrin receptor binding	Interacting selectively and non-covalently with an ephrin receptor	<0.001	0.004
2	Hsa00983	Drug metabolism – other enzymes	<0.001	0.032
3	Hsa00240	Pyrimidine metabolism	0.004	0.032
4	Hsa00460	Cyanoamino acid metabolism	0.007	0.033
5	Growth	The increase in size or mass of an entire organism, a part of an organism or a cell	0.006	0.036
6	Kidney development	The process whose specific outcome is the progression of the kidney over time, from its formation to the mature structure	0.003	0.038
7	Cellular cation homeostasis	The regulation of the levels, transport, and metabolism of cations within a cell or between a cell and its external environment	0.009	0.039
8	Urogenital system development	The process whose specific outcome is the progression of the urogenital system over time, from its formation to the mature structure	<0.001	0.040
9	Lipid biosynthetic process	The chemical reactions and pathways resulting in the formation of lipids	0.006	0.040
10	Regulation of cell growth	Any process that modulates the frequency, rate or extent of cell growth	0.002	0.042
11	Hsa00500	Starch and sucrose metabolism	0.003	0.043
12	Cation homeostasis	The regulation of the levels, transport, and metabolism of cations	0.009	0.044

FDR; false discovery rate.

The seven candidate causal SNPs and 12 candidate causal pathways provided four hypothetical biological mechanisms for GC: (1) rs4745 (non-synonymous coding) and rs12904 (regulatory region) to *EFNA1* gene to ephrin receptor binding; (2) rs1801019 (non-synonymous coding) to *Uridine Monophosphate Synthetase* (*UMPS*) gene to drug and pyrimidine metabolism; (3) rs364897 (non-synonymous coding) to *Glucocerebrosidase* (*GBA*) gene to cyanoamino acid and starch/sucrose metabolism; and (4) rs11187870 (regulatory region), rs2274223 (non-synonymous coding) and rs3765524 (non-synonymous coding) to *Phospholipase C epsilon 1* (*PLCE1*) gene to growth, kidney development, cellular cation homeostasis, urogenital system development, lipid biosynthetic process, regulation of cell growth, and cation homeostasis (Tables 1 and 2).

## Discussion

The results of previous GWAS studies have suggested that the rs227423 at 10q23 (Abnet et al. 2010), rs1336107 at 5q13, rs9841504 at 3q13 (Shi et al. 2011), and rs2976392 and rs2294008 at 8q24 (Sakamoto et al. 2008) SNPs are associated with GC. Although individual GWAS has been successful in finding new susceptibility genes for various complex diseases, none of the GWAS datasets was analyzed to their full potential (Elbers et al. 2009). Individual GWAS data has focused on SNPs with high statistical significance, whereas the other many SNPs have received little attention. Thus, pathway analysis of genomic data with gene set enrichment approach could highlight significant SNPs that have otherwise been hidden during gene- or SNP- based analysis (Ramanan et al. 2012; Elbers et al. 2009).

In this study, we identified seven candidate causal SNPs, four genes, and 12 candidate pathways by ICSNPathway analysis. The candidate SNPs and pathways provided four hypothetical biological mechanisms. In this genome-wide pathway analysis, the most significant GC-associated pathway was that of ephrin receptor binding.

The *EFNA1* (ephrinA1), located within chromosomal region 1q21-q22, is a glycosylphosphatidylinositol (GPI) linkage ligand with a 205-amino acid chain that preferentially binds to the receptor tyrosine kinase EphA2 at sites, where cell-cell contact occurs (Wykosky and Debinski 2008). Some studies have shown an association between GC and *EFNA1*. (Li et al. 2012) reported that rs12904, located in the *EFNA1* gene, is significantly associated with GC risk. (Nakamura et al. 2005) also reported that EFNA1 was overexpressed in 57% of GC tissue samples. Our pathway analysis suggests that the *EFNA1* gene and ephrin receptor binding pathway may play an important role in GC susceptibility.

The *UMPS* is a fundamental enzyme in pyrimidine synthesis (Gusella et al. 2011). A small-scale study in 23 GC patients indicated that *UMPS* polymorphisms are not related to cancer risk in Caucasian GC patients (Gusella et al. 2011). However, further studies are needed to clarify the effects of the candidate *UMPS* gene and *UMPS*-associated pathways on the development of GC.

The *GBA*, located in 1q21-22, encodes a glucocerebrosidase, which catalyzes the hydrolysis of glucocerebroside, a membrane glycolipid, to ceramide and glucose (Velayati et al. 2010). The association of GBA gene mutation with Gaucher’s disease, Parkinson disease, or Lewy body disorder has been reported (Velayati et al. 2010). However, to the best our knowledge, candidate *GBA* and its pathways have not been investigated in GC.

The *PLCE1* gene on chromosome 10q23 encodes an enzyme that catalyzes the hydrolysis of phosphatidylinositol-4,5-biphosphate, generating the secondary messengers inositol 1,4,5-triphosphate and diacylglycerol, which participate in cell growth and differentiation (Bunney et al. 2009). Several studies have reported that the rs2274223 polymorphism in *PLCE1* is a risk factor for GC in the Chinese Han population (Abnet et al. 2010; Zhang et al. 2011b; Wang et al. 2012). In addition, (Luo et al. 2011) suggested that GC patients with this SNP have a survival advantage. However, no significant association was observed between rs2274223 polymorphism and GC in a Caucasian population (Palmer et al. 2012; Kupcinskas et al. 2014).

Previous studies of GC GWAS have suggested that prostate stem cell antigen (*PSCA*) and *MUC1* SNPs are associated with GC risk (Kupcinskas et al. 2014; Rizzato et al. 2013). However, the pathway analysis in our study could not identify these SNPs. This discordance may be caused by several factors, such as ethnic diversity, differences in sample size or type, and variable GWAS array chips.

Several web servers for pathway analysis of GWAS have been offered: *i*-GSEA4GWAS (http://gsea4gwas.psych.ac.cn), VEGAS (https://vegas2.qimrberghofer.edu.au) and DAVID (http://david.abcc.ncifcrf.gov). We selected the ICSNPathway because it is an updated version of *i*-GSEA4GWAS and has advantages in exploring candidate causal SNPs, genes, and disease associations (Zhang et al. 2011a).

The present ICSNPathway analysis has some limitations. First, incomplete annotation of the human genome is an important shortcoming of the pathway-based approach. In addition, imperfect knowledge of their genetic basis in complex diseases may decrease the ability of ICSNPathway analysis to explore true causal SNPs and pathways. Subsequent replication studies are required to confirm the candidate SNPs, genes, and associated pathways (Jia et al. 2011). However, replication in independent datasets is beyond the scope of this study. Pathway analyses using GWAS datasets can be a useful tool in discovering novel genes that are associated with disease susceptibility (Ramanan et al. 2012; Zhang et al. 2011a).

It is rather arbitrary to determine the appropriate threshold for significant SNPs (Ramanan et al. 2012; Lambert et al. 2010). The cut-off levels for significant SNPs have ranged from *P* < 0.05 to *P* < 5 × 10^-8^ (Ramanan et al. 2012). (Lambert et al. 2010) reported that pathway analyses were performed using different levels of cut-off values (<0.01, <0.001, or <0.0001). However, there was little difference in finding significant pathways according to these cut-off values. We adopted *P* value of 1× 10^-4^, considering the number of significant SNPs which could be entered for the pathway analysis.

In conclusion, we carried out ICSNPathway analysis using the GC GWAS data to evaluate genetic associations with GC at the SNP and pathway levels, considering that a pathway-based approach improves the results of individual SNP analyses of GWAS datasets. We identified seven candidate causal SNPs and four genes, and developed four hypotheses that possibly contribute to GC susceptibility. Further studies are needed to confirm and explore genetic variations of the hypothetical pathways underlying GC susceptibility.
